# Identification and Removal of Contaminant Sequences From Ribosomal Gene Databases: Lessons From the Census of Deep Life

**DOI:** 10.3389/fmicb.2018.00840

**Published:** 2018-04-30

**Authors:** Cody S. Sheik, Brandi Kiel Reese, Katrina I. Twing, Jason B. Sylvan, Sharon L. Grim, Matthew O. Schrenk, Mitchell L. Sogin, Frederick S. Colwell

**Affiliations:** ^1^Department of Biology and Large Lakes Observatory, University of Minnesota Duluth, Duluth, MN, United States; ^2^Department of Life Sciences, Texas A&M University Corpus Christi, Corpus Christi, TX, United States; ^3^Department of Biology, The University of Utah, Salt Lake City, UT, United States; ^4^Department of Oceanography, Texas A&M University, College Station, TX, United States; ^5^Department of Earth and Environmental Sciences, University of Michigan, Ann Arbor, MI, United States; ^6^Department of Earth and Environmental Sciences, Michigan State University, East Lansing, MI, United States; ^7^Josephine Bay Paul Center for Comparative Molecular Biology and Evolution, Marine Biological Laboratory, Woods Hole, MA, United States; ^8^College of Earth, Ocean, and Atmospheric Sciences, Oregon State University, Corvallis, OR, United States

**Keywords:** 16S rRNA, contamination, microbial survey, Census of Deep Life, deep subsurface

## Abstract

Earth’s subsurface environment is one of the largest, yet least studied, biomes on Earth, and many questions remain regarding what microorganisms are indigenous to the subsurface. Through the activity of the Census of Deep Life (CoDL) and the Deep Carbon Observatory, an open access 16S ribosomal RNA gene sequence database from diverse subsurface environments has been compiled. However, due to low quantities of biomass in the deep subsurface, the potential for incorporation of contaminants from reagents used during sample collection, processing, and/or sequencing is high. Thus, to understand the ecology of subsurface microorganisms (i.e., the distribution, richness, or survival), it is necessary to minimize, identify, and remove contaminant sequences that will skew the relative abundances of all taxa in the sample. In this meta-analysis, we identify putative contaminants associated with the CoDL dataset, recommend best practices for removing contaminants from samples, and propose a series of best practices for subsurface microbiology sampling. The most abundant putative contaminant genera observed, independent of evenness across samples, were *Propionibacterium*, *Aquabacterium*, *Ralstonia*, and *Acinetobacter*. While the top five most frequently observed genera were *Pseudomonas*, *Propionibacterium*, *Acinetobacter*, *Ralstonia*, and *Sphingomonas*. The majority of the most frequently observed genera (high evenness) were associated with reagent or potential human contamination. Additionally, in DNA extraction blanks, we observed potential archaeal contaminants, including methanogens, which have not been discussed in previous contamination studies. Such contaminants would directly affect the interpretation of subsurface molecular studies, as methanogenesis is an important subsurface biogeochemical process. Utilizing previously identified contaminant genera, we found that ∼27% of the total dataset were identified as contaminant sequences that likely originate from DNA extraction and DNA cleanup methods. Thus, controls must be taken at every step of the collection and processing procedure when working with low biomass environments such as, but not limited to, portions of Earth’s deep subsurface. Taken together, we stress that the CoDL dataset is an incredible resource for the broader research community interested in subsurface life, and steps to remove contamination derived sequences must be taken prior to using this dataset.

## Introduction

From the earliest days of subsurface microbiology research, sample contamination and methods to assess and minimize contamination have been paramount to characterizing the microbiology of these habitats (Phelps et al., 1989). Initially, microbiologists expressed concern about coarse drilling practices that introduced contamination from drilling fluid additives and strategies that focused solely on increased core recovery. While drilling practices are necessary for most subsurface studies, so too is the necessity for clean practices that minimize core contamination. Drilling methods that identify contamination and certify the core (Colwell et al., 1992; Griffin et al., 1997) have and continue to evolve through large drilling efforts such as the International Ocean Discovery Program (IODP) and the International Continental Drilling Program (ICDP). These methods to minimize contamination are now in common use and are routinely employed for microbiology research campaigns (Smith et al., 2000; Kieft et al., 2007; Morono and Inagaki, 2016), and have led to seminal discoveries documenting the extent of subsurface life (Biddle et al., 2006; Orsi W.D. et al., 2013; Inagaki et al., 2015).

With improved drilling practices and new technologies to sample subsurface fluids, a new problem has arisen: the molecular methods used for microbial community characterization and to estimate cell abundance are sensitive enough to detect microbes on the order of a few copies of rRNA genes per sample (Tanner et al., 1998; Hoshino and Inagaki, 2012). This is even true now for microscopic detection of cells with the sensitivity at the level of <10 cells per cm^3^ of sample (Morono and Kallmeyer, 2014). While areas of the subsurface contain high microbial biomass (Inagaki et al., 2015), much of the subsurface has low biomass. For these low biomass environments, the likelihood of contamination from laboratory reagents (i.e., extraction kits, Taq polymerase, or buffers) must be acknowledged (Salter et al., 2014). While standard microbiological sterilization methods are necessary to exclude microbial cells, most of these methods do not eliminate DNA or screen ultra-small cells (<0.2 μm). Thus, it is not unusual to detect cells or evidence of cellular DNA in carefully prepared blank samples (Morono and Inagaki, 2016). The potential for post-core extraction contamination is especially problematic as microbiologists attempt to define the limits of habitability in Earth systems where native biomass is exceedingly low (Inagaki et al., 2015). So even while the need for “clean” drilling strategies and methods of detecting contamination must be sustained and implemented (Wilkins et al., 2014; Friese et al., 2017), we must also scrutinize existing and future datasets to sort out the true representatives of the deep biosphere from imposters represented by contaminating sequences. The development of new approaches for examining materials used during drilling, sampling, and/or DNA extraction and sequencing library preparation is essential. While processing numerous control samples may seem like a Sisyphean task, for low biomass environments, it is indispensable and should be applied to all microbial studies.

To date, studies have focused on limiting field-based contamination (Wilkins et al., 2014; Friese et al., 2017) and the need to control for contamination from molecular reagents (Salter et al., 2014). Here we expand on these studies by focusing on molecular datasets associated with the Census of Deep Life (CoDL), which are derived from diverse subsurface environments and extracted from different labs using several DNA extraction methodologies. The CoDL was established in 2011 through the Deep Carbon Observatory and allowed investigators from around the world to submit DNA for 16S rRNA gene sequencing from deep terrestrial or subseafloor environments. Many of these samples yielded DNA concentrations that were close to the limits of detection. As a legacy database, these sequences are a community resource and as such must be vetted for downstream usage. Here we seek to differentiate DNA sourced from authentic subsurface microbes from that originating due to contamination (e.g., field, lab, or reagent-based contaminants). Furthermore, we identify common sources of contamination within the dataset, methods for identifying and removing contaminants, and finally ways to mitigate contamination when working with low biomass systems.

## Materials and Methods

For this study, two versions of the CoDL dataset were obtained through the Visualization and Analysis of Microbial Population Structure web portal (VAMPS^1^; Huse et al., 2014). The VAMPS web portal allows users to upload data and process using standardized pipelines. Using the VAMPS portal, two datasets were downloaded in May 2017. The first is a taxonomic identification and abundance table of all unique sequences associated with submitted projects (both publicly released and private) and the samples therein. Sample information, which included primer region and DNA extraction method, was downloaded from the accompanying metadata. This dataset, consisting of a total of 460 datasets, was used to assess the extent of contamination using categorical searches based on taxonomic assignments. Due to the diversity of primer sets (both bacteria and archaea specific as well as different variable regions) and sequencing technology (454 pyrosequencing and Illumina) used over the course of the CoDL, clustering sequences from the entire dataset was not possible. For the second dataset, FASTA sequences of only unique, publicly available sequences were downloaded, which consisted of ∼40 million short reads. Again reads were not clustered prior to taxonomic assignment using BLASTn (Altschul et al., 1990). Reads were blasted against the SILVA v128nr database (Pruesse et al., 2007) and for sake of dataset size, only the top blast hit was kept based on bit score and percent identity using a custom perl script (script: postblast.pl^2^).

To highlight current contamination removal tools, a single CoDL study dataset was chosen that was known to have significant contamination and also included a variety of control samples. As this manuscript does not seek to highlight individuals for their contaminated datasets, we have chosen to keep all studies anonymous. BLASTn values for the example study were subsetted from the entire Blast database using R (R Core Team, 2014). SourceTracker2 (Knights et al., 2011) was run using sequenced blank controls as the source of contamination. Oligotyping (Eren et al., 2013) was performed using the VAMPS web portal using default and/or recommended settings.

## Results and Discussion

### Do Contaminants Exist in the Census of Deep Life Dataset?

**FIGURE 1:**
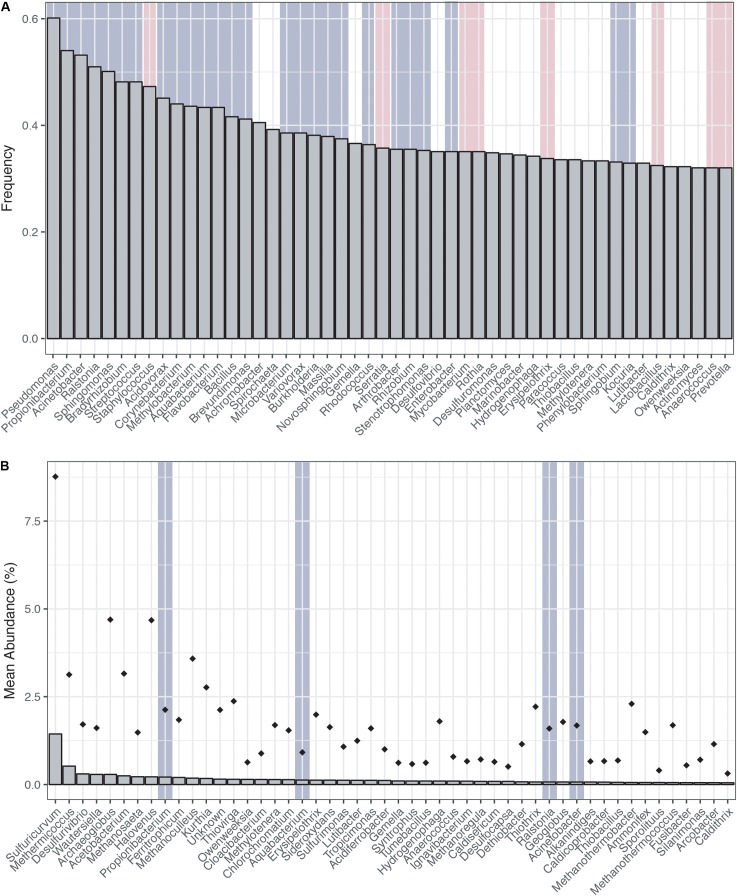
Distribution of top genera from the Census of Deep Life dataset (all publically available data as of May 2017). **(A)** Genera ranked by frequency (left to right, most frequent to least frequent). **(B)** Genera ranked by mean abundance (left to right, most abundant to least abundant). Abundance was calculated on a per sample basis. Diamonds represent the upper maximum standard deviation. Vertical gray highlighting bars indicate genera identified as putative contaminants by previous studies and pink vertical bars represent other contaminants generally associated with the human microbiome.

Ranking the genera most frequently encountered and most abundant (**Figures 1A,B**) across the CoDL dataset, we observe several abundant genera (gray highlight bars) previously identified as potential contaminants in molecular reagents (see **Supplementary Table S1** for full list). When ranked by frequency of occurrence, 17 genera out of 20 were associated with reagent contaminants; of the remainder, one was identified as a potential contaminant and two were not previously listed as contaminants (**Figure 1A**). The top five genera observed were *Pseudomonas*, *Propionibacterium*, *Acinetobacter*, *Ralstonia*, and *Sphingomonas*. Alternatively, when ranking by mean abundance, we also observed the genera *Propionibacterium*, *Aquabacterium*, *Ralstonia*, and *Acinetobacter*. The frequency and abundance of these genera in both ranking methods suggest that when identifying contaminants there are likely two pools to consider: (1) frequently encountered and abundant and (2) frequently encountered and low abundance. In scenario 1, abundance ranking is driven by several samples being highly contaminated, thus driving overall abundance. Whereas in scenario 2, the occurrence of background kit or reagent contaminants is partially suppressed by the sample’s DNA, but because many of these samples are from low biomass environments, the natural microbial DNA cannot completely overcome the kit contamination.

### What Can Sample Blanks (Field and Laboratory) Tell Us?

As Salter et al. (2014) noted, the first line of contamination identification is to incorporate controls. Here, field control samples were based on the individual investigator’s particular experimental design, and may have included drilling fluids, sampling equipment, or blank filters. In the lab, extraction kit, PCR, and sequencing blanks (controls to account for reagent or sample handling contamination) are also necessary to trace the source when contamination may have been introduced. However, when sequencing subsurface samples, great care must be taken when interpreting the data, as these samples should inherently contain extremely low biomass. First, depending on the PCR conditions and reagents used by a lab or sequencing core, amplification of any trace DNA present in the reagents could still occur even at lower thermocycler rounds (<25; Tanner et al., 1998). Second, improper handling during the DNA extraction or the DNA dispersal to PCR plates can occur (Ballenghien et al., 2017). Thus, DNA from actual samples may be aerosolized (Le Rouzic, 2006) and cross-contaminate adjacent wells, including low-biomass control blanks. For the CoDL dataset, testing for aerosolization in control samples is difficult, as most studies did not include controls for sequencing. Third, barcodes can be crossed due to base changes during the amplification or through miscalling due to low quality, leading to reads being counted as controls.

**FIGURE 2:**
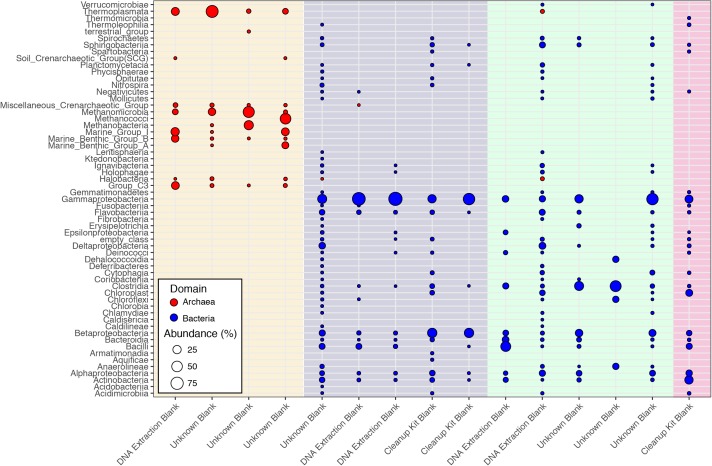
Class-level classifications of sequences associated with control samples from the CoDL. Laboratory controls were included by the primary investigators and were processed with different DNA extraction methods. The colored vertical bands represent primer sets used, from left to right: archaeal V4V5 (cream), bacterial V6 (purple), bacterial V4 (green), and bacterial V4V5 (pink). The diversity of primer sets reflects the different sequencing platforms (454 vs. Illumina) that were used by the CoDL.

While Salter et al. (2014) were able to control and identify contamination in the DNA extraction kits they employed, the DNA provided to the CoDL for sequencing was generated by many labs using a range of DNA extraction methods, which is reflected in the diversity and variability of microbial communities associated sequenced control samples (**Figure 2**). The observed diversity and variability illustrate that removing and controlling for all sequence contamination for the entire CoDL dataset is a difficult task. For the control blanks, the taxonomic breakdown at the class level shows that *Gammaproteobacteria* and *Betaproteobacteria* occur most frequently. However, no consistent taxonomic lineages occurred throughout all the controls (**Figure 1**). If we used the genera in **Supplementary Table S1** as guidelines of “typical” contaminants and apply a strict cutoff for taxonomic-based removal, we would remove ∼27% of the total sequences in the CoDL dataset. Given that we are using taxonomy to identify contaminants, it should be considered that sequence misclassification can occur. Misclassification does occur due to database inconsistencies or due to close resemblance to a known or anticipated contaminant, resulting in removal of “species” that may be ecologically important. In the CoDL dataset, an operational taxonomic unit (OTU)-based approach may be helpful in reducing the dataset size down from millions of sequences to the low thousands. However, this will only work for samples using the same primer set. The effects of misclassification can still be encountered, as it is an issue of classifying the read or representative OTU sequence to a reference database with an algorithm. Given the diversity seen in the blanks, this indicates that when analyzing larger multi-study datasets, contamination removal should be directed on a case-by-case basis at the study level and not the entire dataset.

Interestingly, archaea were observed in both the archaeal and the bacterial V4V5 primer amplified controls (**Figure 1**). The bacterial V4V5 are designed to be highly degenerate and amplify groups from both archaea and bacteria (Bates et al., 2011). While the search for new archaea is intensifying, in general, archaea have received less attention when compared to bacteria, despite their ubiquity and abundance in most environments (Adam et al., 2017). Certainly, in past contamination papers, archaea are not quantified nor are they mentioned as potential contaminants. However, based on this evidence from multiple samples, we suggest that greater examination of archaea as contaminants must be undertaken when investigating subsurface environments or other low biomass environments. Additionally, while contamination from fungi or picoeukaryotes was not analyzed during this study, eukaryotic signatures were observed in the CoDL dataset. We acknowledge their relative importance in the subsurface (Edgcomb et al., 2011; Orsi W. et al., 2013; Rédou et al., 2015) and the need to incorporate this into future CoDL meta-analyses.

### What Is the Source of the Contamination?

**FIGURE 3:**
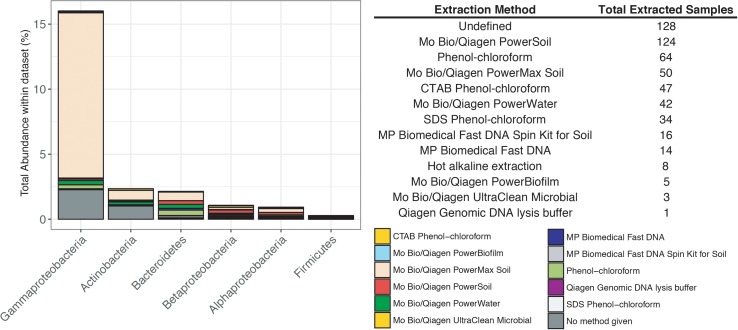
Influence of DNA extraction method on the total abundance of contaminants encountered in the CoDL dataset. The table records the type of DNA extraction method and the total number of samples extracted with that method.

Previous studies have focused on the “kit-ome” or contamination associated with DNA kits (Salter et al., 2014), and while this meta-analysis was not specifically focused on establishing a kit microbiome, the CoDL dataset does provide an unique view of contamination, as samples were processed with multiple DNA extraction methods. As depicted in **Figure 2**, the microbial diversity in sample blanks is both disheartening and intriguing from a methodological standpoint. Members of the *Gammaproteobacteria* were the most frequently encountered contaminants followed by *Actinobacteria*, *Betaproteobacteria*, *Alphaproteobacteria*, *Firmicutes*, and *Bacteroidetes* (**Figure 3**). The most frequently used DNA extraction method among CoDL investigators was the MoBio/Qiagen PowerMax Soil kit. It should be noted that these kits are designed for high biomass, chemically complex samples, and are likely not optimized for low biomass subsurface environments. Recent advancements in portable sequencing technologies like Oxford nanopore^3^ and field-based DNA extractions PureLyse (Claremont BioSolution LLC, Upland, CA, United States) open the possibilities of near real-time identification of microbes in the field. However, in light of molecular and field-based contamination, much care must be taken, especially for identifying *in situ* microorganisms. Additional sources of contamination identified in water purification systems and from human bodies (e.g., researchers performing the extractions) partially overlap with those found in extraction kits (Kulakov et al., 2002; Laurence et al., 2014). However, it is difficult, and in some cases costly, to fully determine the source of contamination (e.g., extraction, water, or researcher). Therefore, we recommend that investigators always include a no-template control with every sequencing run regardless of whether or not a PCR product is visible by gel electrophoresis.

### You Have Been Contaminated, Now What?

As already noted, the notion of contamination removal is not new and several methods have been developed and applied to identify and remove contaminants from 16S rRNA gene datasets, with varying degrees of success. Methods for removal vary from more straightforward hands-on assessment using abundance and frequency of occurrence of OTUs and taxonomic assignments, to using probability algorithms to identify and filter putative contaminants. Below we highlight common methods for removal of contaminants from datasets and use examples from the CoDL dataset to identify putative contaminant sequences. As it is difficult to apply these methods to the entire CoDL dataset, we have focused on a set of samples from a single study that included blanks and exhibits variability in the contamination. We highlight several currently employed techniques to remove contaminating sequences from the data including filtering common contaminants, microbial source tracking (MST), oligotyping, and probability assessment.

### OTU Table Taxonomic and Frequency-Based Filtration

As the simplest form of sequence removal, this method identifies putative contaminants by taxonomic classifications obtained during most OTU clustering pipelines, such as mothur (Schloss et al., 2009) or QIIME (Caporaso et al., 2010). Here sequences that are identified as “common contaminants” are completely removed from downstream analyses prior to calculation of relative abundances. For example, the mothur iTag standard operating procedure designed for analysis of bacterial 16S rRNA gene amplicon datasets recommends that sequences classified as chloroplasts, mitochondria, unknown, Archaea, and Eukarya be removed^4^ (Kozich et al., 2013). However, this is highly dependent on the questions that are being asked. For example, recent work has shown that chloroplast DNA preserved in sediments may be a useful tool for assessing past phototroph communities (Kirkpatrick et al., 2016; Reese et al., 2018). In the case of the mothur SOP, Schloss et al. (2009) typically process gut microbiome samples that contain DNA from both host and degraded food. Because the primer sets that are typically used for microbial community analysis are degenerate, amplification of groups outside of the bacteria and/or archaea is common. Removal of sequences for these common taxonomic groups is necessary. Nonetheless, the introduction of contaminant DNA can happen through several mechanisms, such as inherent properties of the sample (i.e., low biomass), mishandling in the lab, DNA/RNA extraction kit or PCR reagents, or mishandling at the sequencing core. Salter et al. (2014) identified common genera of microorganisms that are typically associated with DNA/RNA kit and molecular biology reagent contaminants (see **Supplementary Table S1**). Removing these putative contaminants from the dataset completely altered the results and downstream interpretations of their data. Thus, identifying and removing these putative contaminants are necessary. This approach has been used for recent deep biosphere investigations (Orsi W.D. et al., 2013; Inagaki et al., 2015; Jørgensen and Zhao, 2016; Labonté et al., 2017; Reese et al., 2018) and has proven effective. In taking this approach, it is important that one includes specific details about how the contaminant subtraction was conducted. This practice will allow other researchers to generate a “clean” dataset from the raw dataset deposited in online repositories for inclusion in meta-analyses.

Whereas bulk taxonomic ID and removal of contaminants may work for some datasets, incorporation of closest relative by BLAST, abundance in each sample, frequency across samples, and the probability of an OTU being real vs. contaminant may also be used. When sequencing blanks have been incorporated, the OTUs present in the controls can be assessed quickly across the entire dataset as being present or absent to gauge the extent to which the real sample may be contaminated (OTU abundances). While this task may be accomplished using spreadsheets, the complexity of next generation datasets is increasingly making the use spreadsheets to analyze datasets obsolete. In addition, the use of spreadsheets is difficult for reproducibility. Thus, we recommend other programs such as R (R Core Team, 2014) or Matlab (Schmidt and Jirstrand, 2005) for efficient processing. When suspicious OTUs are identified by taxonomy (for instance, OTUs belonging to the *Propionibacteria*) and confirmed with BLAST (by looking for highly similar sequences to sequences within one’s own reference database), the frequency of occurrence and abundance of a suspicious OTU is assessed across the entire dataset. This method allows fine-tuning of the removal process and an accounting for the identification of sequences that may be localized to a unique sampling site or geochemical regime. This approach was used to identify sample contamination in hydrothermal plume communities (Breier et al., 2014). As an extension of this strategy, custom scripts can assess the probability of an OTU being a contaminant (Inagaki et al., 2015). In this study, probability-based removal of putative contaminant sequences identified a majority of OTUs as suspicious, resulting in removal of ∼99% of the original datasets for most of their samples.

**FIGURE 4:**
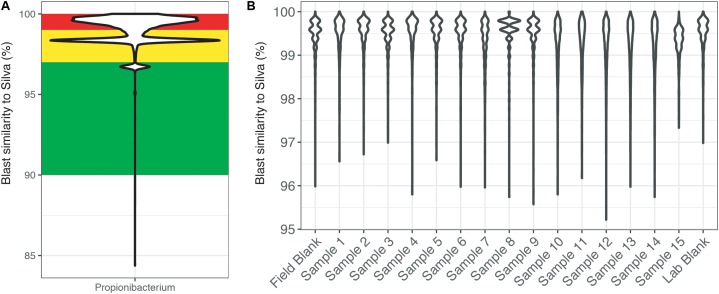
BLAST output of amplicon reads phylogenetically identified to the genus *Propionibacterium* from **(A)** the total CoDL dataset and **(B)** an example CoDL study with previously recognized contamination problems. Violin plots show the distribution of reads and the spread of the data. Amplicon reads were blasted against the SILVA v.127 reference database to identify the closest reference sequence. The top blast hit was chosen based on a combination bit score and percent identity. Reads are classified as being highly suspicious (red = 100–99% similar), putative contaminants (yellow = 99–97% similar), and putative real signal (green = <97% similar).

To highlight this BLAST approach, the genus *Propionibacteria* was chosen because it is commonly associated with human skin, yet it has also been shown to have environmentally relevant metabolisms (Benz et al., 1998; Chang et al., 2011; **Figure 4**). Across the entire dataset, the majority of reads identified as *Propionibacteria* were >97% similar to sequences within the SILVA database (**Figure 4A**). Additionally, this figure highlights the secondary problem associated with taxonomy-based removal. The long tail of the violin plots shows that some reads are very dissimilar (<96% similarity) and do not taxonomically belong to the *Propionibacteria* genus. Misidentification of short sequencing reads is not a new topic (Wang et al., 2005) and is associated with the 16S rRNA gene region being sequenced and sequence reads being divergent from reference database entries. Furthermore, additional error could be accumulated with scripts written to choose the best candidate sequence. Thus, using taxonomic only approaches to remove sequence reads may also remove taxonomically novel reads from potentially important microorganisms that have not yet been tabulated in genomic databases. In our example dataset (**Figure 4B**), all of the *Propionibacteria* reads were >96% similar to sequences in the SILVA database. This highlights an instance where complete taxonomic removal of these sequences would be substantiated.

### Microbial Source Tracking

Microbial source tracking is an investigative strategy for identifying sources of elevated concentrations, commonly used in the public health and food industries (Scott et al., 2002). MST can be applied here to track contaminating sequences from extraction or environmental blanks. Available software such as SourceTracker2 (Knights et al., 2011) uses a Bayesian sampling approach to estimate the proportion of contaminants in a given community that come from possible source environments. Alternatively, commands such as get.coremicrobiome within mothur (Schloss et al., 2009) or compute_core_microbiome.py in QIIME (Caporaso et al., 2010) allow a researcher to look for commonalities across all samples. When paired with a control sample, these tools are able to quickly identify the potential contaminants within datasets. Furthermore, depending on the types of controls, one could identify when the contamination occurred – i.e., during sampling, pre-extraction processing, reagents, or sequencing. Ideally, several controls must be sequenced alongside DNA from environmental samples, and in the case of SourceTracker2 (a follow-up version of Source Tracker; Knights et al., 2011), it is necessary to have a potential contamination source sample for the program to generate putative contaminant OTUs. Alternatively, if no blanks are available, then get.coremicrobiome (mothur) or compute_core_microbiome.py (QIIME) can identify OTUs that are common to all samples. While ubiquity does not imply contamination, coupled to taxonomic identification and environmental chemistry, one can begin to infer if contamination is possible especially if the environmental samples come from disparate sites.

**FIGURE 5:**
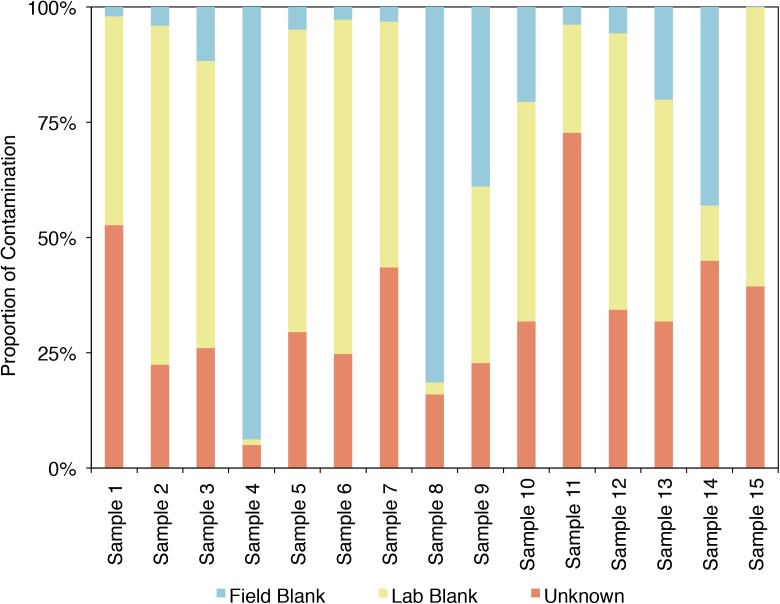
Proportion of the bacterial community of an example CoDL study with previously recognized contamination problems that can be attributed to given sources, as determined by SourceTracker 2 (Knights et al., 2011).

SourceTracker2 was used to analyze the example CoDL dataset that included multiple blank samples and is suspected of contamination, also used in **Figures 4**, **5**. A large proportion, ranging from 25 to 95% of the bacterial communities from each sample in this study likely contains contaminants, either from the field or laboratory. The proportion of the community from an “unknown” source (represented in orange) could be the true signal of the sample or it could come from a potential contaminant not tested. Therefore, it is important, particularly when dealing with precious, low-biomass samples, to collect as many control samples as possible. Using a tool like SourceTracker2 can help provide confidence in a dataset or identify which samples warrant downstream analyses.

### Single Nucleotide Resolving “Oligotyping”

Operational taxonomic unit clustering at typical “species level” threshold of 97% is highly contentious in the field of microbial ecology (Janda and Abbott, 2007). While building OTUs helps reduce the complexity of the data by lumping sequences into bins meant to approximate species, this practice may promote problems such as decreased taxonomic resolution with shorter reads (Wang et al., 2005) and OTU species inflation (Knapp et al., 2005). The recent emergence of oligotyping, which resolves populations at the sub-OTU level by quantifying the abundance of single nucleotide variants within a traditional OTU cluster, is now being applied to whole communities. Previous work has shown that this method could be used to determine the probable origin of key contaminant microbes in wastewater (McLellan et al., 2013) and more recently to resolve population structures of potential cyanobacteria populations (Berry et al., 2017). With regard to tracking sources of contamination, oligotyping contaminants may be useful for separating real from contaminant taxa or to track contaminants in individual reagent components.

**FIGURE 6:**
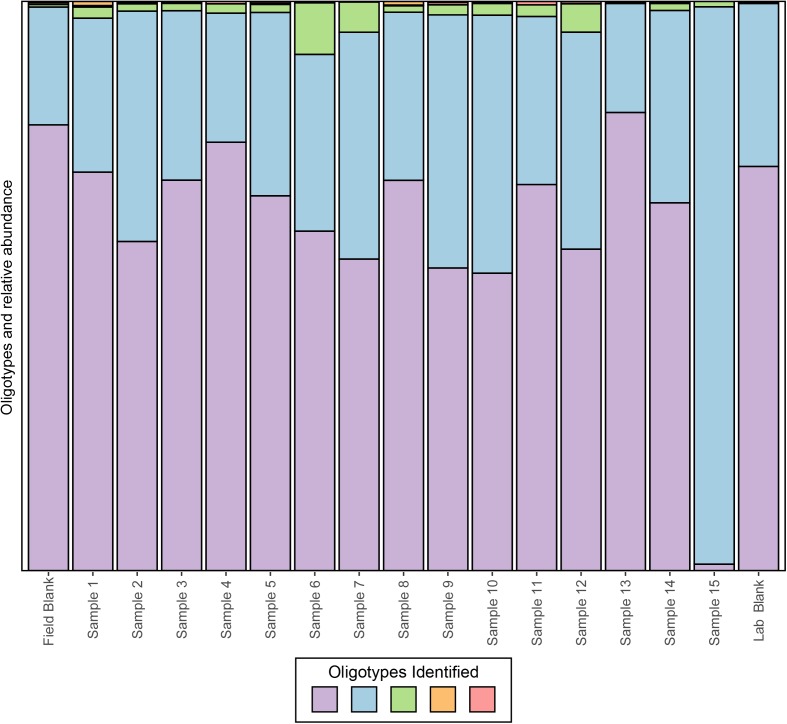
Oligotyping of sequences associated with the dominant OTU identified to genus *Propionibacterium* from an example CoDL study with previously recognized contamination problems. Oligotype variants are identified by color and the size of the bar represents the relative proportion in each sample.

To date, several methods have been developed, however, we will focus on applying oligotyping (Eren et al., 2013), which is integrated into the VAMPS (Huse et al., 2014) online web portal that hosts the CoDL datasets. Using the same test CoDL dataset with known sequencing blanks and significant contamination, we have applied oligotyping to identify the inter-sequence variability within the most abundant *Propionibacterium* OTU, a skin associated bacterium and commonly identified contaminant. Here, we show that within this out, a total of five oligotypes were identified, two dominant (purple and light blue) and three rare (green, orange, and pink; **Figure 6**). These results are interesting but also are difficult to interpret in terms of identifying whether the source of contamination occurs from sample handling in the field or during the extraction process. The high similarity of the lab blank to field blanks and field samples suggests that kit contamination is likely responsible for the presence of the two dominant oligotypes. The presence of the rare oligotypes in the field blank and the samples collected appears to have occurred from handling in the field. The power for elucidating contamination at sub-OTU resolution is yet another tool for the modern microbiologist. However, we stress that as with all of these techniques, use of only a single approach will likely not be sufficient because all methods have strength and weaknesses. Here, the presence of several variants within the single OTU shows the presence of many potential strains but this does not provide information as to the nucleotide similarities between the sequences. Depending on the OTU clustering method, the cutoff threshold and how similar sequences are to one another will naturally vary. Thus, using a secondary method to understand how similar these sequences are to one another would give more insight into the nature of the oligotypes observed.

### Best Practices to Prevent Contamination Throughout Your Experiment

Before starting any microbiological study, particularly those based on low-biomass environments, we recommend considering both quality assurance (QA) and quality control (QC) during sample collection, processing, and analysis. This approach assures that each study is of highest quality, reproducible, and gives the greatest confidence in any resulting data.

**FIGURE 7:**
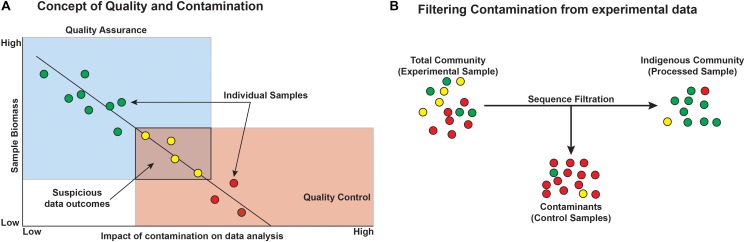
Conceptual outcomes of contamination and the general removal of contaminants from datasets. **(A)** Microbial contamination and its impacts on the study are a function of microbial biomass, such that with decreasing biomass, the likelihood and impact of contamination increase and must be considered when designing the experiment. Colors represent minimal (green), some (yellow), and high (red) contamination signal. **(B)** Filtering contaminants from existing datasets can be aided by sequencing control blank samples, ideally from each step of the experiment. Circles represent individual operational taxonomic units (OTUs) or phylotypes and the colors represent green = taxa that are authentic to the environment, yellow = suspicious taxa, and red = contaminant taxa.

*Quality assurance* is defined as *process* oriented and focuses on contamination *prevention* during all stages of sample collection (**Figure 7**). Before beginning a deep microbiology sample collection effort, the drilling team must develop a plan that includes contamination prevention strategies that are consistent with the environment that will be sampled, the method of sampling, and the post-extraction sample handling and shipping. The first step in core QA is the assessing the integrity of the core barrel and the core itself. This first step identifies whether samples are likely to be compromised by the drilling fluid. Review of available logs, core descriptions, and CT scans may reveal fractures in rock that could result in drill fluid penetration. Drill fluid intrusion may be quantified through the addition of perfluorocarbon tracers (Lever et al., 2006), but methods incorporating inexpensive particulate dyes are now available for terrestrial drilling projects (Friese et al., 2017). Additional tracers have been developed and tested; examples include chemical tracers, fluorescent microspheres, iodine, ink, artificial oligonucleotides, perfluorocarbons, salmon DNA, or even a specific microorganism not expected to be detected at the sampling site such as *Bacillus nigricans* (Smith et al., 2000; Kieft et al., 2007; Cardace et al., 2013; Morono and Inagaki, 2016; Friese et al., 2017; Orcutt et al., 2017). Collecting and testing the drilling lubricants before, during, and after core collection allow researchers to trace the presence of the fluid (indicating intrusion) and also provide a background microbial community from which contaminating sequences may later be removed *in silico* (Struchtemeyer et al., 2011; Struchtemeyer and Elshahed, 2012; Inagaki et al., 2015). Again, drilling fluid composition is quite diverse, and researchers may opt to use sterile water as the basis of the drilling fluid (Cardace et al., 2013), although this is not a guaranteed method of removing contamination and does limit the sources of contamination. This is feasible for land-based drilling studies (such as ICDP drilling projects and WIZZARD in Antarctica), but not for seafloor drilling, where the very large volumes of required drilling fluid precludes the use of sterile water. In cases where deep subsurface microbiological samples are collected without the use of drilling (i.e., subsurface wells, mines, and caves), special precautions should be considered. For example, collecting surrounding “service” water (water used in mining activities), groundwater, or formation fluid is necessary to determine the background microbial community in order to discern the indigenous community of interest (Kieft et al., 2007). Additionally, fluid sampling requires the use of sterile filter housings, tubing, and collection bottles. Filters flooded with preservation fluids must also be sterile.

Once the core or water is collected, additional QA procedures should be implemented during processing to control contamination. In some cases, it may be useful to design a sampling program that includes “blind” controls which can be distributed to labs that are carrying out the analyses. Examples of “blind” samples are those that may be sterilized in the field or spiked with a known microbe prior to packaging and shipping. Inclusion of these blind samples and subsequent analysis may aid in determining the factors responsible for sample alteration (e.g., changes that may have occurred during sample shipping). If possible, samples should be obtained from the center of the core (i.e., subsample plugs) and from the center of a long core section (i.e., far from the core ends that were physically cut). Processing the core should take place in a lab setting with limited sources of contamination during this vulnerable stage of the process. Steps to control the sterility of the environment may include limiting access to the area, HEPA filtering the air, decontaminating surfaces (e.g., gamma irradiation, UV, bleach, and ethanol), using personal protective equipment (e.g., hair nets, face masks, shoe covers, and cleanroom suits), and generally following aseptic technique while processing (Sanders, 2012; Morono and Inagaki, 2016).

*Quality control* is a necessary system of maintaining standards, through operational techniques that allow us to compare genetic data from other studies. This step is *product* oriented and focuses on contamination identification. In order to determine if a sample contains potentially contaminating sequences, we must first identify what the contamination is and its origin. Contamination can be tracked by collecting and analyzing the background microbiome surrounding the sample collection site (e.g., surrounding environment and drill fluid), the destination laboratory or DNA extraction site (e.g., air, bench, and hood), ultrapure water systems, the reagents or kits used during extraction (referred to herein as a “blank”), and potentially the laboratory researchers performing the extractions. Additionally, processing multiple sub-samples from the same core or fluid as well as extracting DNA from a representative microbial community (i.e., mock community) may be extracted or sequenced alongside the samples to assess recovery, thus acting as an internal standard. Although blanks should yield little or no genetic material, it is important to perform their sequence analyses alongside the samples of interest.

Once sequences are obtained from real samples, blanks, and background, *in silico* techniques can be applied to identify endemic microbial community from the contaminating microorganisms. It should be noted that *in silico* removal requires researchers to make assumptions about what is real and what is not, and the best situation is to minimize this process, where possible through stringent QA practices (see **Figure 7B** for conceptual diagram). As such, sampling subsurface environments is difficult, expensive, and often cannot be repeated. Thus, any data generated through these studies are important and need to be incorporated into current and future studies of the subsurface. However, as we have outlined collection practices change through time and by laboratory, thereby increasing the complexity of contamination sources and the likelihood that contaminating sequences exist in the dataset.

### Conclusion and Future Directions

**FIGURE 8:**
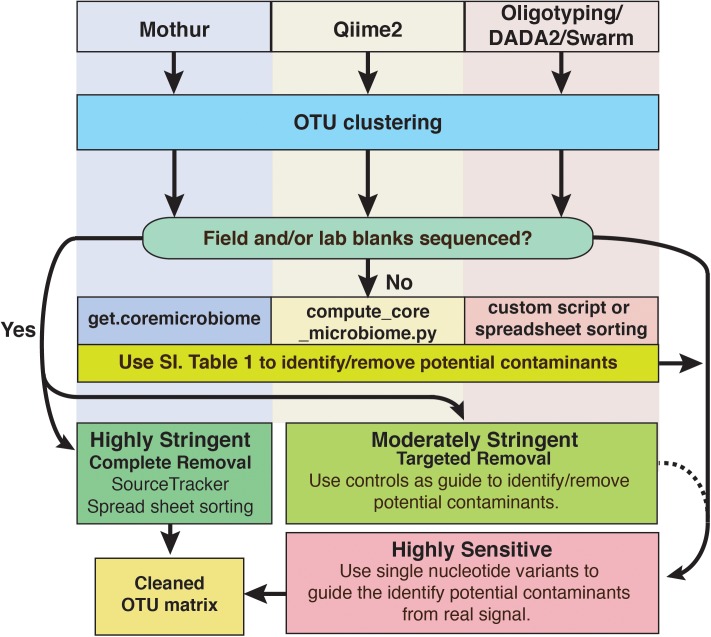
Schematic diagram of options to identify and remove contaminant amplicon reads from sequencing datasets. Because several options are available and are being developed continually, we suggest these methods are not comprehensive and depending on the dataset may not be suitable. However, we hope that this serves as a guideline for approaching contamination removal.

For deep subsurface microbiology studies, adherence to best QA/QC practices is essential and can include approaches that are decades old or recently introduced. Even using the most assiduous techniques, contamination would seem to be an inevitable outcome of modern microbiome studies of subsurface environments and other low biomass settings, as the sensitivity of modern sequencing platforms continues to increase. However, it does not have to ruin investigations. To cope with this problem, we have outlined a putative workflow for identifying and removing contaminating amplicon reads from next generation sequencing datasets (**Figure 8**). To reiterate, we recommend that every study of deep subsurface habitats incorporates controls into each step of the process: sample collection, extraction, and sequencing. Resulting reads from the samples and controls should be clustered according to the individual’s preferred algorithm. From here, more than one approach of removing the contaminating sequences may be necessary. As a first pass, it is a good idea to put the OTUs through a program that identifies overlaps between samples and field or sequencing blanks. We acknowledge that this approach alone is fallible in that putative contaminants may not be a contaminant at all since OTU creation with short read technology is at a finer resolution than the taxonomic identification, which is limited to the genera level. Once the putative contaminants are identified, oligotyping may be used for resolving sequence variability. Through this approach, we showed that at the genera level, some sequences related to *Propionibacterium* would have been removed, perhaps unnecessarily based on BLAST results.

To date, studies identifying contamination from reagents and kits have primarily focused on bacteria. However, sequencing efforts by the CoDL have shown that archaea and eukarya (fungi and picoeukaryotes) were also present in extraction blanks. The presence of methanogenic archaea in sample blanks from the deep subsurface is striking, as these groups are very important to carbon cycling in many subsurface environments as well as in human gastrointestinal tract. Therefore, as researchers probe for life in these low biomass environments, analyzing these data for biogeochemically important organisms must be scrutinized. We strongly recommend that amplicon sequencing incorporates archaeal and eukaryotic primer controls as needed.

As the fields of geomicrobiology and microbial ecology increasingly incorporate shotgun metagenomic sequencing and advanced genome binning methods, researchers have the ability to identify contaminants by their genomes rather than a single gene such as the 16S rRNA gene. As we have discussed above, the use of 16S rRNA genes to identify contaminants has many caveats and there is no standardized way of approaching data contamination. However, by using the entire genome, the ability to specifically identify contaminants from real signal is greatly enhanced, especially when sequencing control blanks along with the real sample. High pH serpentinite environments are often dominated by *Betaproteobacteria* (Schrenk et al., 2013), and if taxonomy of a single marker gene alone, such as the 16S rRNA gene, was used to identify contamination, these bacteria might be flagged as potential contaminants. However, through the use of metagenomics (Brazelton et al., 2012), researchers have shown that members of the *Betaproteobacteria* are in fact ubiquitous and ecologically important members of alkaline (pH > 10) serpentinite environments, which was subsequently verified by culture-dependent studies (Suzuki et al., 2014). Additionally, Olm et al. (2017) recovered *Delftia* genomes from soil metagenome assemblies and were able to show these genomes were from contamination using comparative genomics. Thus, the ability to trace contamination with high precision using techniques that identify strain level variation, gene additions or loss, and the presence of plasmids is at the forefront of this detective work.

The deep subsurface, despite being the largest biome on Earth, is still vastly under sampled, especially when compared to shallow subsurface environments such as soil. Thus, one of the main legacies of the CoDL is the data (i.e., 16S ribosomal RNA gene sequences) generated from these unique subsurface environments. By providing a window to the deep biosphere, future subsurface scientists have unprecedented access to data to generate testable hypotheses regarding how life thrives in these environments. However, as we have highlighted here, care must be taken when analyzing these data from low biomass subsurface environments. As we push the boundaries of life discovery on Earth and other planets, these recommendations also apply and ring true. We encourage researchers who examine environments where biomass is at a minimum to live by the mantra “quality in, quality out” during the entire process from collecting the samples to processing the sequencing data. Remembering that contamination can come from anywhere helps to ease the analysis and interpretation of these intriguing datasets.

## Author Contributions

CS, BR, KT, JS, and FC conceived the manuscript, analyzed the data, and wrote the manuscript. SG provided the data and analyzed the portions of the dataset. MOS and MLS conceived the manuscript and wrote the manuscript. CS and BR performed equal shares of the manuscript preparation and share first authorship.

## Conflict of Interest Statement

The authors declare that the research was conducted in the absence of any commercial or financial relationships that could be construed as a potential conflict of interest.

## Footnotes

1 https://vamps2.mbl.edu

2 https://github.com/Geo-omics

3 https://nanoporetech.com/

4 https://mothur.org/wiki/MiSeq_SOP

## Supplementary Material

TABLE S1 A list of common contaminants previously identified as putative contaminants.
