# Supplementary material for: Identification and Removal of Contaminant Sequences From Ribosomal Gene Databases: Lessons From the Census of Deep Life
Source: Front Microbiol. 2018 Apr 30;9:840. doi: 10.3389/fmicb.2018.00840 (PMC5945997; doi:10.3389/fmicb.2018.00840)
Supplement: TABLE S1 — A list of common contaminants previously identified as putative contaminants. [file Table_1.pdf]

| Phylum | Class | List of constituent contaminant genera | Extraction | Water | Microbiome |
|---|---|---|---|---|---|
| Proteobacteria | Alphaproteobacteria | *Afipia* | | | |
| | | *Aquabacterium* | | | |
| | | *Asticcacaulis* | | | |
| | | *Aurantimonas* | | | |
| | | *Beijerinckia* | | | |
| | | *Bosea* | | | |
| | | *Bradyrhizobium* | | | |
| | | *Brevundimonas* | | | |
| | | *Caulobacter* | | | |
| | | *Craurococcus* | | | |
| | | *Devosia* | | | |
| | | *Hoefleae* | | | |
| | | *Mesorhizobium* | | | |
| | | *Methylobacterium* | | | |
| | | *Novosphingobium* | | | |
| | | *Ochrobactrum* | | | |
| | | *Paracoccus* | | | |
| | | *Pedomicrobium* | | | |
| | | *Phyllobacterium* | | | |
| | | *Rhizobium* | | | |
| | | *Roseomonas* | | | |
| | | *Sphingobium* | | | |
| | | *Sphingomonas* | | | |
| | | *Sphingopyxis* | | | |
| | | *Acidovorax* | | | |
| | | *Azoarcus* | | | |
| | | *Azospira* | | | |
| | | *Burkholderia* | | | |
| | | *Comamonas* | | | |
| | | *Cupriavidus* | | | |
| | | *Curvibacter* | | | |
| | | *Delftiae* | | | |
| | | *Duganella* | | | |
| | | *Herbaspirillum* | | | |

| | | | | | |
|---|---|---|---|---|---|
| | Betaproteobacteria | *Janthinobacterium* | ■ | | |
| | | *Kingella* | ■ | | |
| | | *Leptothrix* | ■ | | |
| | | *Limnobacter* | ■ | | |
| | | *Massilia* | ■ | | |
| | | *Methylophilus* | ■ | | |
| | | *Methyloversatilis* | ■ | | |
| | | *Neisseria* | | | ■ |
| | | *Oxalobacter* | ■ | | |
| | | *Pelomonas* | ■ | | |
| | | *Polaromonas* | ■ | | |
| | | *Ralstonia* | ■ | ■ | |
| | | *Schlegelella* | ■ | | |
| | | *Sulfuritalea* | ■ | | |
| | | *Undibacterium* | ■ | | |
| | | *Variovorax* | ■ | | |
| | Gammaproteobacteria | *Acinetobactera* | ■ | | |
| | | *Enhydrobacter* | ■ | | |
| | | *Enterobacter* | ■ | | |
| | | *Escherichia* | ■ | | |
| | | *Nevskia* | ■ | | |
| | | *Pasteurella* | | | ■ |
| | | *Pseudomonas* | ■ | | |
| | | *Pseudoxanthomonas* | ■ | | |
| | | *Psychrobacter* | ■ | | |
| | | *Stenotrophomonas* | ■ | | |
| | | *Xanthomonas* | ■ | | |
| Acidobacteria | | *Predominantly unclassified Acidobacteria Gp2* | ■ | | |
| | | *Aeromicrobium* | ■ | | |
| | | *Actinomyces* | | | ■ |
| | | *Arthrobacter* | ■ | | |
| | | *Beutenbergia* | ■ | | |
| | | *Brevibacterium* | ■ | | |
| | | *Corynebacterium* | ■ | | ■ |
| | | *Curtobacterium* | ■ | | |

| | | | | | |
|---|---|---|---|---|---|
| Actinobacteria | | *Dietzia* | | | |
| | | *Geodermatophilus* | | | |
| | | *Janibacter* | | | |
| | | *Kocuria* | | | |
| | | *Microbacterium* | | | |
| | | *Micrococcus* | | | |
| | | *Microlunatus* | | | |
| | | *Patulibacter* | | | |
| | | *Propionibacterium* | | | |
| | | *Rhodococcus* | | | |
| | | *Tsukamurella* | | | |
| Bacteroidetes | | *Chryseobacterium* | | | |
| | | *Dyadobacter* | | | |
| | | *Flavobacterium* | | | |
| | | *Hydrotalea* | | | |
| | | *Niastella* | | | |
| | | *Olivibacter* | | | |
| | | *Parabacteroides* | | | |
| | | *Pedobacter* | | | |
| | | *Prevotella* | | | |
| | | *Wautersiella* | | | |
| Deinococcus-Thermus | | *Deinococcus* | | | |
| Firmicutes | | *Abiotrophia* | | | |
| | | *Bacillus* | | | |
| | | *Brevibacillus* | | | |
| | | *Brochothrix* | | | |
| | | *Facklamia* | | | |
| | | *Lactobacillus* | | | |
| | | *Paenibacillus* | | | |
| | | *Ruminococcus* | | | |
| | | *Staphylococcus* | | | |
| | | *Streptococcus* | | | |
| | | *Veillonella* | | | |
| Fusobacteria | | *Fusobacterium* | | | |
